# Supplementary material for: Continuous Material Deposition on Filaments in Fused Deposition Modeling
Source: Polymers (Basel). 2024 Oct 15;16(20):2904. doi: 10.3390/polym16202904 (PMC11511081; doi:10.3390/polym16202904)
Supplement: Supplementary file 1 [file polymers-16-02904-s001.zip › polymers-3180721-supplementary.pdf]

**Continuous Material Deposition on Filaments in Fused Deposition Modeling – Supporting Information**

Guy Naim, Shlomo Magdassi* and Daniel Mandler*

Institute of Chemistry, the Hebrew University of Jerusalem, Jerusalem 9190401, Israel

Daniel.mandler@mail.huji.ac.il

## Supplementary Table

The following printing parameters (Table S1) were applied using the slicer program for the various samples produced. The diameter of the coated filament fed into the program was based on a caliber measured diameter of 1.79 ± 0.04 mm.

| | |
|---|---|
| Filament diameter | 1.80* mm |
| Nozzle Temp - first layer | 215 °C |
| Nozzle Temp - other layers | 210 °C |
| Bed Temp (all layers) | 60 °C |
| Layer height (including the first layer) | 0.2 mm |
| Solid layers (Top and Bottom) | 3 |
| Infill type | Rectilinear for dogbones and aligned cuboids<br>Gyroid for disk shapes |
| Infill density | 100% for dogbones and aligned models<br>15% for disk shapes |
| Ironing | Enabled for disks only<br>15% Flow rate, 0.1 mm spacing between passes |

**Table S1.** Printing parameters used for all samples.
* The diameter of PLA filament used for non-coated samples was 1.75 mm.

## Supplementary Figure

The two orientations printed for mechanical testing, as presented in Figure S1, were chosen to test the strength of possible prints when stress is applied, for more details see section 3.2.

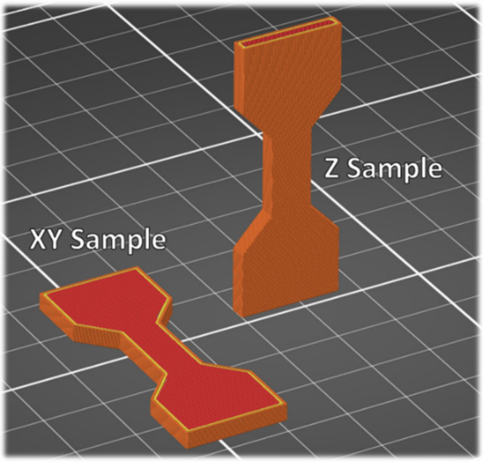


**Figure S1.** The two orientations were printed for tensile tests. XY samples were printed face-down on the heat bed, testing the stress acting on the printed mesh. Z samples were printed to test the strength of the layer adhesion.

**Detection of ciprofloxacin in polylactide acid 3D-printed samples**

Determining the weight ratio of Ciprofloxacin (Cip) in polylactide acid (PLA) 3D-printed samples was conducted based on the spectroscopic method (Fig. S2A). Firstly, the PLA samples were dissolved in 5 ml acetonitrile (ACN) at 65 °C, achieving a clear and colorless solution. A volume of 15 ml of hydrochloric acid (HCl) 0.1 M was slowly added to the solution. While Cip shows great solubility in aqueous acidic conditions, PLA tends to aggregate, partially sink, and partially float in the medium. Once the solution stabilized after at least 15 undisturbed minutes (Figure S2B), the middle part of the suspension was spectrophotometrically measured.

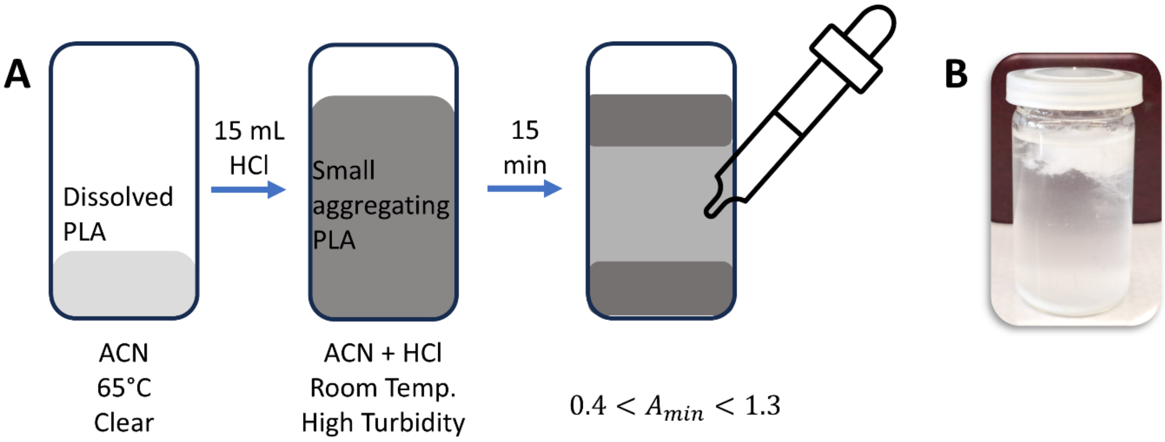


**Figure S2.** (A) A schematic representation of the dissolution and subsequent recombination of the tested polylactide acid (PLA) samples, showcasing the low turbidity medium between the floating and the aggregated PLA particles after reaching equilibrium. The conditions or turbidity for each step are as stated. (B) A picture of a solution at equilibrium before extraction and absorbance testing.

The absorbance of the solution was tested over a wide range of wavelengths (Figure S3) to reduce the turbidity of the medium. The difference between the absorbance maximum and plateau showed linear dependency and clear repetativity (Figure S4). The calibration solutions were prepared by first dissolving uncoated printed PLA and adding known volumes of Cip solution 200 µg/mL in HCl 0.1 M mixed in the 15 mL of added HCl solution.

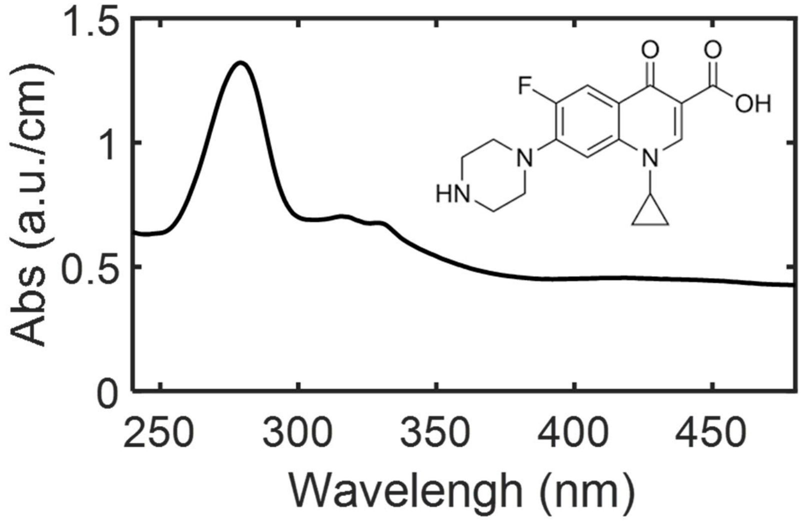


**Figure S3.** A measured absorbance spectrum for a printed sample made with a coating solution including 20 mg/ml PLA and 10 mg/ml Ciprofloxacin (Cip).

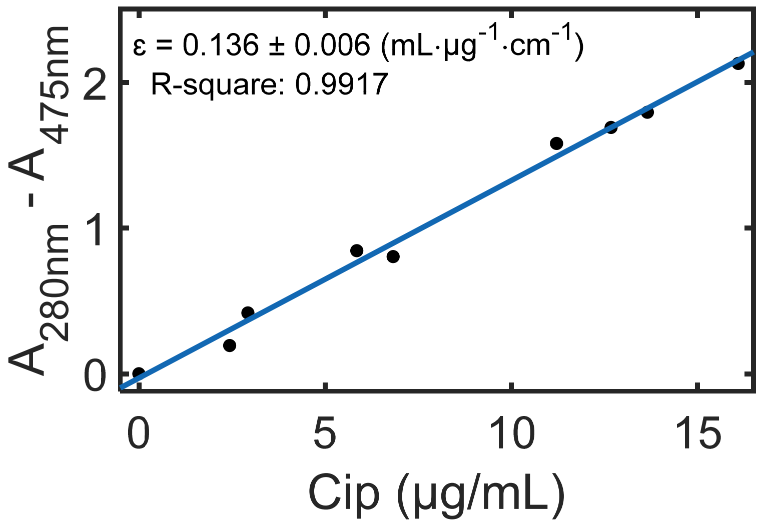


**Figure S4.** Beer-Lambert calibration curve based on the absorbance difference of Cip between 280 nm and 475 nm.

### ZnO NPs stability in the coating solution

ZnO nanoparticles (NPs) suspension inside the Dichloromethane/Tetrahydrofuran (DCM/THF) coating solution is generally unstable. The NPs rapidly aggrege and sink without stirring after cooling down to room temperature. However, PLA showed stabilizing properties to ZnO NPs in the coating solution, creating a usable coating solution for our purposes (Figure S5).

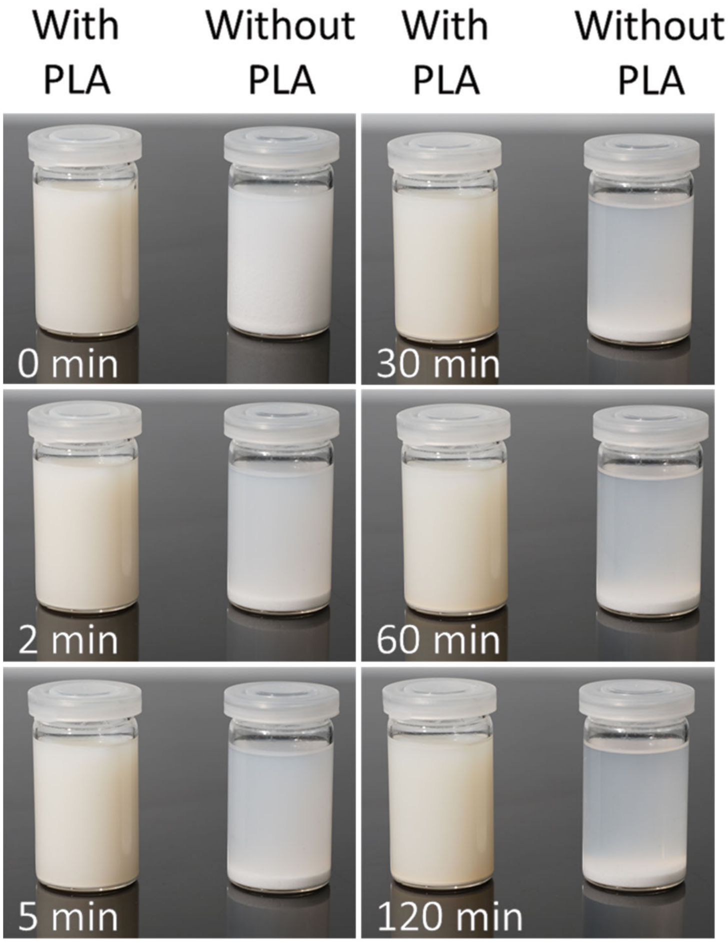


**Figure S5.** ZnO nanoparticles (NPs) in Dichloromethane/Tetrahydrofuran (DCM/THF) solutions, with and without dissolved PLA, over time.

The solution without PLA showed fast separation and sinking of the ZnO NPs, creating a white powder pile at the bottom of the vial. The solution containing PLA started slowly showing separation after around one hour, focused on the topmost and bottom of the suspension. Despite this separation, no noticeable changes to the turbidity of the solution were observed.

The major time difference between the times in which the changes occurred and the clarity of only the solution where no PLA was present, obvious stabilizing forces must act between the ZnO NPs, the polymer, and the hydrophobic solution.

**Cip release model**

The release profile of the antibiotics in an aqueous solution was fitted according to the Ritger-Peppas equations and empirical model for diffusion (eq. S1-S2).[1-3]

$$\frac{M_t}{M_\infty} = k \cdot t^n \qquad \text{(eq. S1)}$$

$$\log\left(\frac{M_t}{M_\infty} \cdot 100\%\right) = \log(k) + n \cdot \log(t) \qquad \text{(eq. S2)}$$

Where $M_t$ and $M_\infty$ are the amount of drug released to the Phosphate-buffered saline (PBS) by time $t$ and the total amount of drug in the sample, respectively. $k$ is the incorporating characteristics constant of the macromolecular network system and the drug, and $n$ is the exponent constant of the transport mechanism, coupling a Fickian and a non-Fickian diffusion mechanism. The model matches the results closely as seen in Figure S6. and Table S2., and the three required conditions of the "perfect sink" model are met. Firstly, the concentration of Cip in the solution remains negligible compared to the drug solubility in water. Secondly, as determined by the experimental model, the total release of the drug remains below 60% of the total amount in the sample. Lastly, no major degradation or mass loss of the bulk materials occurred within 35 days of incubation, showing a maximum mass reduction of 0.24%.

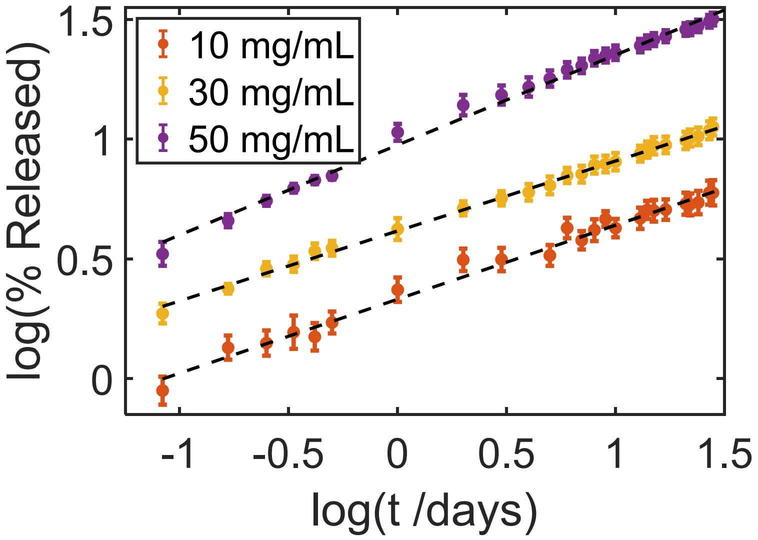


**Figure S6.** Accumulating relative release of Cip from samples coated with a coating solution of 20 mg/mL PLA and 10-50 mg/mL dissolved Cip. Release profiles were fitted following eq. S2 (dashed black lines).

| | 10 mg/mL | 30 mg/mL | 50 mg/mL |
|---|---|---|---|
| $n$ | 0.309 ± 0.007 | 0.292 ± 0.003 | 0.376 ± 0.006 |
| $\log(k)$ | 0.332 ± 0.007 | 0.617 ± 0.003 | 0.976 ± 0.006 |
| $R^2$ | 0.9894 | 0.9973 | 0.9935 |

**Table S2.** Fitting parameters for eq. S2 as presented in Fig. S6.

The high accuracy of the model to the measurements points out that PLA layers function as an inert and rate-controlling membrane. The release rate suggests that the major denominators of the diffusion are the thickness and permeability of the coating. These types of kinetics are often optimal for drug delivery, as unforeseen changes in the environments have a lesser effect on the devices, producing useful for a mid/long-term administration of medication.[4]


1. Siepmann, J.; Peppas, N.A. Higuchi equation: Derivation, applications, use and misuse. *International Journal of Pharmaceutics* **2011**, *418*, 6-12, doi:10.1016/j.ijpharm.2011.03.051.
2. Ritger, P.L.; Peppas, N.A. A simple equation for description of solute release II. Fickian and anomalous release from swellable devices. *Journal of Controlled Release* **1987**, *5*, 37-42, doi:10.1016/0168-3659(87)90035-6.
3. Fu, Y.; Kao, W.J. Drug release kinetics and transport mechanisms of non-degradable and degradable polymeric delivery systems. *Expert Opinion on Drug Delivery* **2010**, *7*, 429-444, doi:10.1517/17425241003602259.
4. Yang, W.-W.; Pierstorff, E. Reservoir-Based Polymer Drug Delivery Systems. *SLAS Technology* **2012**, *17*, 50-58, doi:10.1177/2211068211428189.
